# A Study of the Information Embedding Method into Raster Image Based on Interpolation

**DOI:** 10.3390/jimaging8100288

**Published:** 2022-10-19

**Authors:** Elmira Daiyrbayeva, Aigerim Yerimbetova, Ivan Nechta, Ekaterina Merzlyakova, Ainur Toigozhinova, Almas Turganbayev

**Affiliations:** 1 Institute of Information and Computational Technologies Committee of Science of the Ministry of Education and Science of the Republic of Kazakhstan, Almaty 050010, Kazakhstan; 2 Institute of Automation and Telecommunication, Academy of Logistics and Transport, Almaty 050012, Kazakhstan; 3 Institute of Automation and Information Technologies of the Satbayev University, Almaty 050013, Kazakhstan; 4 Department of Applied Mathematics and Cybernetics, Siberian State University of Telecommunications and Information Science, Novosibirsk 630102, Russia

**Keywords:** LSB steganography, interpolation, secret message, RS analyze, image

## Abstract

This article is devoted to the study of the improved neighbor mean interpolation (INMI) steganographic method. To date, no steganalysis of such a method of information embedding has been carried out. We implemented the INMI method of embedding messages in raster files and conducted a stegoanalysis on a set of 800 images of 225 × 225 size. Experimentally, we found that with this embedding method, the maximum container capacity is 21% and that it depends on the contents of the container. It is established that only 60 files out of 800 actually have the maximum capacity. We presented the calculation of the Type I error and the percentage of information detection in the obtained containers by the regular–singular (RS) method. The results showed that the considered steganographic algorithm is resistant to RS steganalysis and is comparable to the stegosystem of the permutation method investigated in one of our previous articles.

## 1. Introduction

To ensure the security of a communication channel, messages transmitted between two subscribers are modified in such a way that their interception by a third party is impossible. Usually, such modification utilizes cryptography methods. In a general setting, the message is encoded with the help of some secret key, accessible only to the sender and the recipient. Receiving the original message from the encoded one is almost impossible without knowing the secret key. Accordingly, the analysis of data transmitted over an open communication channel does not allow a third party to freely read the original message [1].

Steganography studies the confidential communication methods and is intended to hide the very existence of communication, unlike cryptography, which aims to make communication incomprehensible to those who do not have the necessary keys [2].

The problem of steganographic analysis or stegoanalysis is an important component of building an integrated information security system. First of all, steganographic methods of information transfer are employed for hidden data transfer in various files carrying redundant data. In this vein, media files are typically used as containers. Here, image files are of the greatest interest for research since images are exchanged much more often compared to other types of media, e.g., audio. For example, users actively send images to each other in various instant messengers and social networks [3].

In a general setting, a message can be confidentially transmitted via containers such as text, audio, video, image, and an executable file (program). Embedding a message into text can be carried out by replacing synonyms [4]. Here, words in the source text are replaced with synonyms corresponding to the embedded message. As a result, the obtained text has the same meaning but already contains a hidden message.

Embedding messages into images is a common practice in steganography. Any picture is represented as a matrix of pixels, where each pixel is defined via an RGB triple. One way of embedding a message here is the so-called least significant bit (LSB) embedding method [5,6]. In this method, the least significant bit of a pixel’s color is replaced with a secret message. The use of this approach does not distort the visual perception of the image.

The rapid development of LSB embedding methods has given rise to the emergence of steganalysis methods for images, i.e., methods for detecting the fact of transmission of a secret message. To fight against detection, LSB injection is carried out not in all pixels but only in a subdomain of the image, and these pixels are selected in a pseudo-random manner.

## 2. Literature Review and Problem Statement

In this paper, we are considering a steganographic method using interpolation. The essence of interpolation is to use the available data to obtain expected values at unknown points.

Image interpolation is a very important branch in image processing and is widely used in the world of imaging, e.g., in 3D medical imaging to compensate for the lack of information in image reconstruction by modeling additional images between acquired 2D images [7].

In [8], Ki-Hyun Jung demonstrated a new interpolation method in data hiding. The proposed method of neighbor mean interpolation (NMI) is characterized by low time complexity and high computational speed, which is important when working with large images. The experimental results of the NMI algorithm show that the proposed method can embed a large number of secret data while maintaining very high visual quality where the peak signal-to-noise ratio (PSNR) is guaranteed to be higher than 35 dB, which is an excellent rate for a reversible data concealment method. Similarly, this method has the greatest capacity among other reversible data hiding ones and is comparable to other data hiding methods.

Yevsyutin et al. made an overview of the main algorithms for embedding information into digital images using interpolation and proposed the INMI algorithm’s modification. The overview also included a comparison of the algorithms in terms of PSNR and maximum capacity [9].

As indicated in [9], all of the algorithms for embedding information into digital images using interpolation are constructed similarly. The input is an M×N-sized image and a secret message representing a binary sequence. The stegocontainer image with the size M×N is formed from the original image using an interpolation algorithm.

Following [9], let us consider two interpolation methods: NMI and INMI.

First, let us focus on the NMI method. In this algorithm, image interpolation is carried out in the following form: let I(i,j) be the pixel value of the original image, then the pixel value of the container image C(i,j) will be calculated in the following form (Equation (1)):

(1) $$C(i,j)=\left\{\begin{array}{l} I(i,j)ifi=2m,j=2n,\\ (I(i,j-1)+I(i,j+1))/2,ifi=2m,j-2n+1,\\ (I(i-1,j)+I(i+1,j))/2,ifi=2m+1,j-2n,\\ (I(i-1,j-1)+C(i-1,j)+C(i,j-1))/3,else, \end{array}\right.$$

where $m=0,1,\ldots \frac{M}{2}-1,n=0,1,\ldots ,\frac{N}{2}-1$.

The pixel values of the image are calculated as follows. For each of the disjoint blocks with a size of 2 × 2 pixels, it is required to find the value of $d_{k}$ by the formula (Equation (2)):

(2) $$d_{k}=C(i,j)-C_{d}$$

where $C_{d}$ is the upper-left pixel of the block, $k=\bar{1,3}$, after which the number of $n_{k}$ bits that can be embedded in the block and its integer representation $b_{k}$ is calculated, and the values of the corresponding pixels of the image are calculated.

Compared to INMI, the embedding capacity of NMI is less, while the quality of the resulting stegoimage is higher.

Now, let us consider the modified INMI method presented in [9]. The method is based on the use of the Lagrange interpolation polynomial of the second degree to obtain the container image. The image obtained by adding additional rows and columns of pixels to the original image is considered as 5-pixel fragments, numbered from 0 to 4. In this setting, the pixel values of the container image are calculated as follows (Equation (3)):

(3) $$C_{k}=C_{0}\frac{\left(x_{k}-2)\cdot (x_{k}-4\right)}{8}+C_{2}\frac{x_{k}(x_{k}-4)}{-4}+C_{4}\frac{x_{k}(x_{k}-2)}{8},$$

where $k=\bar{1,3}$ is the pixel number in a fragment of five pixels.

As shown in [9], the modified INMI method yields increase in the PSNR value with a slight decrease in the embedding capacity. In this article, we performed a stegoanalysis of the modified INMI method using some known algorithms. In general, this type of stegoanalysis can be applied to the whole class of interpolation methods.

## 3. Materials and Research Methods

This article aims to investigate the considered modification of the INMI method by conducting a steganalysis with available means. We use RS analysis as the primary method of image steganalysis in this study. This type of steganalysis allows us to obtain results comparable to the findings of Merzlyakova [10]. RS analysis is based on the application of double statistics obtained from spatial correlations in images.

Currently, there are no studies analyzing the performance of such algorithms using stegoanalysis methods.

One of the preeminent methods of statistical stegoanalysis is the regular–singular (hereinafter referred to as RS) method, which was developed by Fridrich et al. in 2001 [11,12].

The RS analysis method serves to detect LSB embedding and uses a sensitive method of dual statistics derived from spatial correlations in the input images. In a general LSB picture, the matrix can be predicted to some extent from the matrices of the remaining 7 bits. Such a prediction becomes less reliable after embedding information in the least significant bits, as LSBs become random. This can be mathematically expressed and used to construct a sensitive and accurate stegoanalysis method. For high-quality images taken from a digital camera or scanner, RS analysis indicates that the safe capacitance is less than >0.005 bits per pixel.

The essence of the method is as follows: the entire image is divided into groups of n pixels $G(x_{1},x_{2},\ldots ,x_{n})$, where *n* is even. For groups of pixels, the regularity function f(G) is defined. By pixel value, we mean a number from 0 to 255.

(4) $$f(x_{1},x_{2},\ldots ,x_{n})=\sum {}_{i=1}^{n-1}\left|x_{i+1}\right.-\left.x_{i}\right|$$

The F(x) function is called flipping and has the F(F(x))=x property. Two flipping functions are defined: $F_{1}$, corresponding to the inversion of the least significant bit of the pixel, and $F_{-1}$, which is the inversion with the transfer to the most significant bit:

(5) $$F_{1}=\left\{\begin{array}{l} x+1,0\leq x\leq 254\\ 0,x=255 \end{array}\right.\;\mathrm{and}\;F_{-1}=\left\{\begin{array}{l} x-1,0\leq x\leq 255\\ 255,x=0 \end{array}\right.$$

When flipping is applied to a group, a transformed group of pixels is obtained. All groups of pixels are divided into classes as follows:

If f(F(G)>f(G), then f∈R(regular);
If f(F(G)<f(G), then f∈S(singular);
If f(F(G)=f(G), then f∈U(unusable).

The RS method may indicate a small non-zero message length due to random deviations even for an empty container. This initial non-zero deviation can be either positive or negative and imposes a limit on the achievable accuracy of the RS analysis.

For each group, flipping is performed twice: with a direct and with an inverted mask. After carrying out classification operations for all groups, a number of quantitative characteristics are calculated:

- The number of regular mask groups $M:R_{M}$;
- The number of unusual groups for the mask $M:S_{M}$;
- The number of regular groups for the inverse mask $-M:R_{-M}$;
- The number of unusual groups for the inverse mask $-M:S_{-M}$.

RS analysis shows more accurate results for messages that are distributed around the image, compared to the analysis of messages that are concentrated in a certain area of the image.

**Figure 1:**
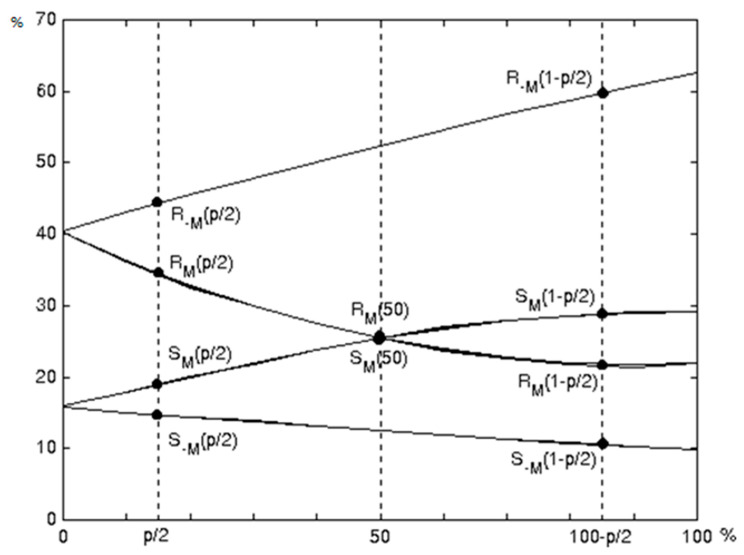
RS–diagram.

Figure 1 shows a typical RS-plot of $R_{M},S_{M},R_{-M},S_{-M}$ values versus the number of pixels with inverted LSB in the image (See Figure 1).

Merzlyakova proposed methods for embedding messages in BMP files [10] and carried out their RS analysis, which helped identify the stability of the various embedding algorithms.

## 4. Research Results

### 4.1. Conducting RS Analysis

To evaluate the modified INMI algorithm, we use the RS method offered by Yeltysheva et al. [13]. The maximum amount of information that can be written to a container using the injection method is called the empirical capacity of the container. The parsing program of RS analysis yields the amount of embedded information (L) as a percentage of the empirical capacity of the container, which is calculated as in LSB embedding.

$$C_{LSB}=3wh\;\mathrm{bit}$$

**Figure 2:**
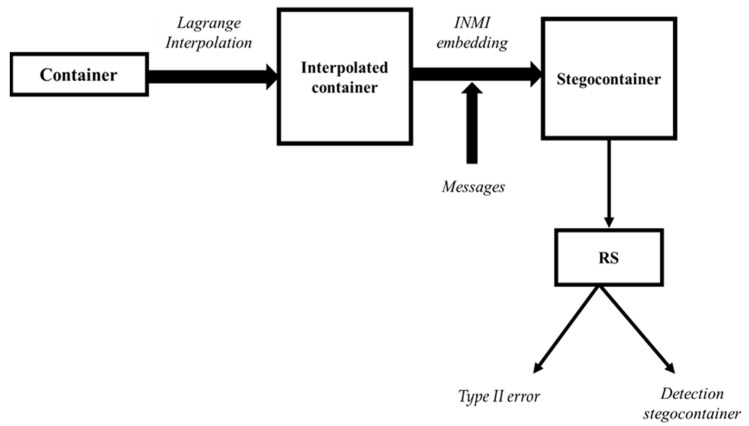
Scheme of experimental studies.

By the value of *L*, one can judge whether the container is full or empty: at L ≥ 5%, RS classifies the container as full. We also mention two hypotheses: $H_{s}$, meaning that the container contains a stegomessage, and an alternative hypothesis $H_{s}$, meaning that the container does not contain embedded information. The decision rule is that each container is assigned one of two hypotheses. Two types of errors are possible here: Type I error, which consists in establishing the hypothesis $H_{s}$ when the container is empty, and Type II error, when the decision $H_{s}$ is made when there is embedded information in the container. The scheme of experimental studies presented below shows the obtained results (See Figure 2).

To conduct an RS analysis of this method, we needed to determine the capacity of containers in terms of the percentage of involvement of the least significant parts of the image matrix. It is paramount to compare the obtained results of steganalysis with the results of other embedding methods considered by Merzlyakova [10].

Based on the modified INMI algorithm, we determine that the maximum container capacity is 21% and that it depends on the image contents.

After conducting a study on a set of 800 images of 225 × 225 size [14], we concluded that 60 of them have the maximum capacity. The findings presented in Table 1 display the RS analysis Type I errors on empty containers. In contrast with Table 1, from the results of the RS analysis in Table 2, one can conclude that the method is resistant to the RS analysis.

The findings of the stegoanalysis presented in Table 1 and Table 2 indicate that the percentage of embedded information detection using the proposed method is approximately equal to the percentage of files with Type I errors.

Accordingly, Table 3 and Table 4 present the results of the study of 60 pictures from the full set of 800 images, with an average filling percentage of 12% (See Table 3 and Table 4).

Apparently, the less information we embed in the image, the less likely it is that detectable features will appear as a result of the implementation process [15]. As we see from the tables above, the modified INMI method is resistant to an RS attack as much as the stegosystem of the permutation method for raster images, described in [10]. The percentage of change of the lower bits in the compared methods is approximately the same and differs in different implementations of the permutation stegosystem [10]. Since the lower bits are not the only ones involved in the interpolation method, it makes sense to also analyze the compression ratio of empty and filled containers.

Consequently, sustainable implementation methods using interpolation are crucial for further development. Therefore, we plan to perform and study implementation methods using the Bezier curve.

### 4.2. Compression Ratio Analysis

It is acknowledged that the original container and the information added to it are statistically independent; therefore, when hidden data are added to the container, its size during compression increases compared to the size of the compressed initial empty container [10]. Changes in the compression ratio in the opposite direction also indicate signs of change. Let us consider the study in which we will determine the compression ratios of empty and filled containers. The compression ratio is the main characteristic of the compression algorithm. It is defined as the ratio of the number of original uncompressed data to the number of compressed data.

Table 5 and Table 6 show the results of comparing the compression ratios for empty and filled containers. Here, $X=\{x_{1},\ldots x_{N}\}$ is the sequence of data bytes of the empty container, and f(X,n) is the compression ratio of the sequence X by the ZIP archiver. $Y=\{y_{1},\ldots y_{N}\}$ is a sequence of data bytes of the filled container, f(Y,n) is the compression ratio of the *Y* sequence by the RAR archiver, and δ(X,n) is the difference between the compression ratios of the segments of the *X* and *Y* sequences. For convenience, the part of the results are presented apart in the Tables below since they are monotonous for the entire selection of files (See Table 5 and Table 6).

## 5. Conclusions

There are various studies in the field on the topic of developing new algorithms for embedding information into graphics files, but it is the interpolation methods that we think have been incompletely investigated and need to be clarified. Our goal was to find out how the proposed methods are resistant to stegoanalysis.

The novelty of the proposed research is that image steganography methods using interpolation have not been previously subjected to stegoanalysis, and the studies cited in the literature do not reflect the stego-resistance of these methods in any way. Our studies help to determine the level of stegoanalysis robustness of the proposed methods.

We have implemented and investigated the notable INMI algorithm, or rather its best modification, and carried out stegoanalysis of this embedding method. The results obtained can be compared with the stegoanalysis of the methods considered by Merzlyakova.

Based on the examined INMI algorithm, we dispose that the maximum container capacity is 21% and depends on the image. Based on the results of a study on a set of 800 225 × 225 images, we determine that 60 of them have the maximum capacity. The above presented findings display the calculation of the Type I errors as 0%.

The findings of RS analysis are shown in Table 2, where one may observe that the method is stable RS and comparable in resistance and capacity to the stoichiometry of the method of permutation for raster images, considered in the above mentioned studies. Additionally, the percentage of change of low-order bits in the compared methods is approximately the same and differs in different implementations of the stegosystem. Nevertheless, because the interpolation method involves not only low-order bits, we used a specific method to analyze the degree of compression in empty and filled containers.

The results of stegoanalysis were obtained on a set of photos with a certain amount of embedded information. If this amount is too small (less than 5% of the total data), the RS cannot be applied. Additionally, the input data for embedding must not have dependencies but must be a random sequence of bits, so the correlations used in the stegoanalysis method are not broken. The methods used are suitable for bmp raster images.

The above result can be used in further developments of information embedding methods using interpolation, to compare the effectiveness of embedding methods on attributes such as stego-resistance.

Likewise, in the study, we determined the compression ratios of empty and filled containers. The value of the differences in the coefficients δ(X,n) obtained in this study is close to zero, which means the statistical structures of the empty and filled containers are similar.

Thence, we plan to develop sustainable steganographic methods for embedding information into raster files using other interpolation tools, as well as to analyze them using different approaches.

## Figures and Tables

**Table 1:** RS analysis on a set of 225 × 225 empty containers.

	*L*		
	0%	1–4%	5% and More
Percentage of correctly detected stegocontainers	54	46	-

**Table 2:** RS analysis on a set of 225 × 225 containers filled with 21% interpolation method.

	*L*		
	0%	1–4%	5% and More
Percentage of correctly detected stegocontainers	53	47	-

**Table 3:** RS analysis on a set of 225 × 225 empty containers (800 images).

	*L*		
	0%	1–4%	5% and More
Percentage of correctly detected stegocontainers	61	39	-

**Table 4:** RS analysis on a set of 225 × 225 containers filled using the interpolation method by 12%.

	*L*		
	0%	1–4%	5% and More
Percentage of correctly detected stegocontainers	51.5	48	-

**Table 5:** Comparison of differences in compression ratios for empty and filled containers.

File Name	f(X,n), MB	f(Y,n), MB	δ(X,n)
001.BMP	0.133	0.134	0.02
002.BMP	0.137	0.138	0.01
003.BMP	0.086	0.086	0.00
004.BMP	0.105	0.106	0.01
005.BMP	0.151	0.153	0.00
006.BMP	0.033	0.033	0.00
007.BMP	0.146	0.148	0.02
008.BMP	0.125	0.126	0.01
009.BMP	0.117	0.118	0.01
010.BMP	0.072	0.072	0.00
117.BMP	0.065	0.065	0.00
140.BMP	0.059	0.059	0.00
180.BMP	0.129	0.131	0.02
222.BMP	0.134	0.135	0.01
244.BMP	0.102	0.103	0.01
25.BMP	0.135	0.136	0.01
250.BMP	0.072	0.072	0.00
258.BMP	0.129	0.131	0.02
317.BMP	0.071	0.072	0.01
37.BMP	0.133	0.134	0.01
388.BMP	0.108	0.109	0.01
405.BMP	0.086	0.086	0.00
465.BMP	0.132	0.134	0.02
521.BMP	0.098	0.099	0.01
528.BMP	0.117	0.118	0.01
610.BMP	0.100	0.100	0.00
68.BMP	0.150	0.152	0.02
72.BMP	0.105	0.107	0.02
752.BMP	0.147	0.149	0.02
785.BMP	0.114	0.115	0.01
796.BMP	0.069	0.069	0.00
Average value	0.108	0.109	0.01

**Table 6:** Comparison of compression ratio differences for empty and filled containers (by RAR archiver).

File Name	f(X,n), MB	f(Y,n), MB	δ(X,n)
001.BMP	0.099	0.101	0.02
002.BMP	0.104	0.107	0.03
003.BMP	0.068	0.069	0.01
004.BMP	0.088	0.090	0.02
005.BMP	0.114	0.117	0.03
006.BMP	0.029	0.029	0.00
007.BMP	0.110	0.114	0.04
008.BMP	0.091	0.094	0.03
009.BMP	0.089	0.091	0.02
010.BMP	0.058	0.058	0.00
117.BMP	0.049	0.049	0.00
140.BMP	0.041	0.041	0.00
180.BMP	0.093	0.096	0.03
222.BMP	0.091	0.093	0.02
244.BMP	0.078	0.080	0.02
25.BMP	0.089	0.091	0.02
250.BMP	0.060	0.060	0.00
258.BMP	0.099	0.102	0.03
317.BMP	0.056	0.057	0.01
37.BMP	0.101	0.104	0.03
388.BMP	0.074	0.076	0.02
405.BMP	0.066	0.067	0.01
465.BMP	0.100	0.103	0.03
521.BMP	0.071	0.073	0.02
528.BMP	0.087	0.089	0.02
610.BMP	0.085	0.087	0.02
68.BMP	0.112	0.115	0.03
72.BMP	0.076	0.079	0.03
752.BMP	0.108	0.112	0.04
785.BMP	0.079	0.080	0.01
796.BMP	0.057	0.058	0.01
Average value	0.082	0.083	0.01

## Data Availability

Not applicable.
